# Lipofilling in Breast Oncological Surgery: A Safe Opportunity or Risk for Cancer Recurrence?

**DOI:** 10.3390/ijms22073737

**Published:** 2021-04-03

**Authors:** Francesca Piccotti, Ilona Rybinska, Elisabetta Scoccia, Carlo Morasso, Alessandra Ricciardi, Lorena Signati, Tiziana Triulzi, Fabio Corsi, Marta Truffi

**Affiliations:** 1 Laboratorio di Nanomedicina ed Imaging Molecolare, Istituti Clinici Scientifici Maugeri IRCCS, 27100 Pavia, Italy; francesca.piccotti@icsmaugeri.it (F.P.); carlo.morasso@icsmaugeri.it (C.M.); alessandra.ricciardi@icsmaugeri.it (A.R.); 2 Molecular Targeting Unit, Department of Research, Fondazione IRCCS Istituto Nazionale dei Tumori, 20133 Milan, Italy; ilona.rybinska@istitutotumori.mi.it (I.R.); tiziana.triulzi@istitutotumori.mi.it (T.T.); 3 Breast Unit, Surgery Department, Istituti Clinici Scientifici Maugeri IRCCS, 27100 Pavia, Italy; elisabetta.scoccia@icsmaugeri.it (E.S.); fabio.corsi@icsmaugeri.it (F.C.); 4 Dipartimento di Scienze Biomediche e Cliniche “L. Sacco”, Università Degli Studi di Milano, 20157 Milano, Italy; lorena.signati@unimi.it

**Keywords:** lipofilling, breast reconstruction, adipose tissue, adipose-derived stromal cells, breast cancer, oncological safety, breast cancer recurrence, pre-clinical studies

## Abstract

Lipofilling (LF) is a largely employed technique in reconstructive and esthetic breast surgery. Over the years, it has demonstrated to be extremely useful for treatment of soft tissue defects after demolitive or conservative breast cancer surgery and different procedures have been developed to improve the survival of transplanted fat graft. The regenerative potential of LF is attributed to the multipotent stem cells found in large quantity in adipose tissue. However, a growing body of pre-clinical evidence shows that adipocytes and adipose-derived stromal cells may have pro-tumorigenic potential. Despite no clear indication from clinical studies has demonstrated an increased risk of cancer recurrence upon LF, these observations challenge the oncologic safety of the procedure. This review aims to provide an updated overview of both the clinical and the pre-clinical indications to the suitability and safety of LF in breast oncological surgery. Cellular and molecular players in the crosstalk between adipose tissue and cancer are described, and heterogeneous contradictory results are discussed, highlighting that important issues still remain to be solved to get a clear understanding of LF safety in breast cancer patients.

## 1. Introduction

Lipofilling (LF), also named autologous fat transplantation (AFT) or fat grafting (FG), is a reconstructive and esthetic technique that is increasingly being used in the field of breast reconstructive surgery [1]. Nowadays, it is widely used to promote an improved reconstruction of the breast after mastectomy or breast-conserving surgery (BCS). However, important questions have arisen about the risk of breast cancer (BC) relapse for patients undergoing a LF in the area of the previous BC treatment, especially after conservative treatment, leading to a skeptical or cautious approach by many surgeons to this technique [2,3,4]. Evidence from in vitro studies has revealed that LF could act fueling BC [5,6,7,8,9,10,11,12], though this assumption has not been replicated or supported in clinics [13]. Therefore, concerns remain over the oncological safety of LF and its effect on cancer recurrence. This review aims to provide an integrated overview of both the clinical and the pre-clinical approaches to the suitability of LF after breast surgery in oncological patients, pointing out the several questions still unsolved and the aspects that mainly should be investigated to step toward a clear understanding of risks vs. safety of LF for breast oncological application.

## 2. Lipofilling of the Breast

### 2.1. History and Surgical Practice

LF was first used for filling defects and remodeling body shape about a century ago [14], but its routine use dates back to the early 1980s, when, following the advent of liposuction, large amounts of unwanted fat could be removed from different body areas using small access incisions and a suction cannula. Bircoll first described a method that coupled liposuction with autologous transplantation of the harvested fat in the breast [1]. The highlighted advantage for this new technique was the presence of virtually limitless donor tissue that was soft and malleable to be used for simple, esthetic augmentation of the breast, correction of breast asymmetry, correction of breast deformities, as an adjunct or primary tool in breast reconstruction, or for soft tissue coverage of breast implants [1]. Bircoll’s contribution at beginning was sharply criticized leading to a negative position statement about the procedure released by the American Society of Plastic Surgeons (ASPS) in 1987. In 2007, Coleman gave a fundamental turning point by publishing a review of 17 breast augmentation and reconstruction patients treated using AFT and then followed up with serial photography [15]. After him, a few European surgeons persisted and continued to push for applying this technique, not for cosmetic augmentation only [16,17,18]. For BC surgery, the LF procedure brings advantage in different situations as correction of defects and asymmetry following wide local excision (or BCS), with or without radiotherapy, improvement of soft tissue coverage following implant-based breast reconstruction, volume replacement of implants in unsatisfactory oncoplastic breast reconstruction outcomes, augmentation of volume and refinement after autologous breast reconstruction, and whole breast reconstruction with serial FG and scar corrections.

Different techniques for fat harvesting and LF have been described, but in all of them, autologous fat is harvested, processed, and grafted [19]. Prior to surgery, the different fatty areas of the body are carefully examined to identify natural fat deposits. The most commonly used donor site is the abdominal one because it is usually one of the largest fat deposits. Other possible sites are the region of the greater trochanter (saddle bags) and the inside of the thighs and knees [20]. Several techniques have been proposed for fat collection, to understand which method allows to harvest more viable and functional adipocytes. A commonly used method for fat harvesting is based on vacuum aspiration or aspiration with syringe (“dry” technique). The “wet” technique differs because it is based on the injection of a tumescent fluid consisting of NaCl, adrenaline, and a local liquid anesthetic drug (Klein solution) [21]. It has been proved that the extent of trauma to the collected fat influences the integrity of the adipocytes and a reduced pressure stress increases vitality and improves graft survival [19], though cell viability in dry and wet samples did not appear to differ [22]. However, the “dry” technique can lead to an increased demand for analgesic drugs to avoid patient discomfort [19,20,21,22]. To minimize the damage of adipocytes, a technique has been described by using a 3-mm, blunt-edged, 2-hole cannula connected to a 10-mL syringe, while fat is suctioned manually by withdrawing the plunger [23]. Higher graft viability with lipoaspirates is obtained using a 6-mm cannula rather than a 4-mm or 2-mm cannula [24]. It has been reported that suction-assisted lipoaspiration (SAL) with 0.5-bar negative pressure leads to cell yield and viability comparable to manual lipoaspiration [25]. New methods of lipoaspiration have been recently developed, such as power-assisted, laser-assisted, and ultrasound-assisted liposuction (UAL), for achieving rapid tissue harvest, promoting skin tightening, and minimizing harvest-site morbidity [26]. UAL is becoming more popular and frequently used as it improves the process of lipoaspiration, by decreasing blood loss and tissue trauma [27]. During UAL, a specialized probe or cannula transmits ultrasound vibrations into the fat tissue [26], leading to emulsification of the fat, making it easier to be harvested [28]. The regenerative abilities of adipocytes harvested via a third-generation UAL device compared with those obtained via standard SAL seems to be equal, whereas laser-assisted liposuction, which uses thermolysis to selectively lyse adipocytes, has been shown to decrease adipocytes yield and viability compared with SAL [29].

**Figure 1:**
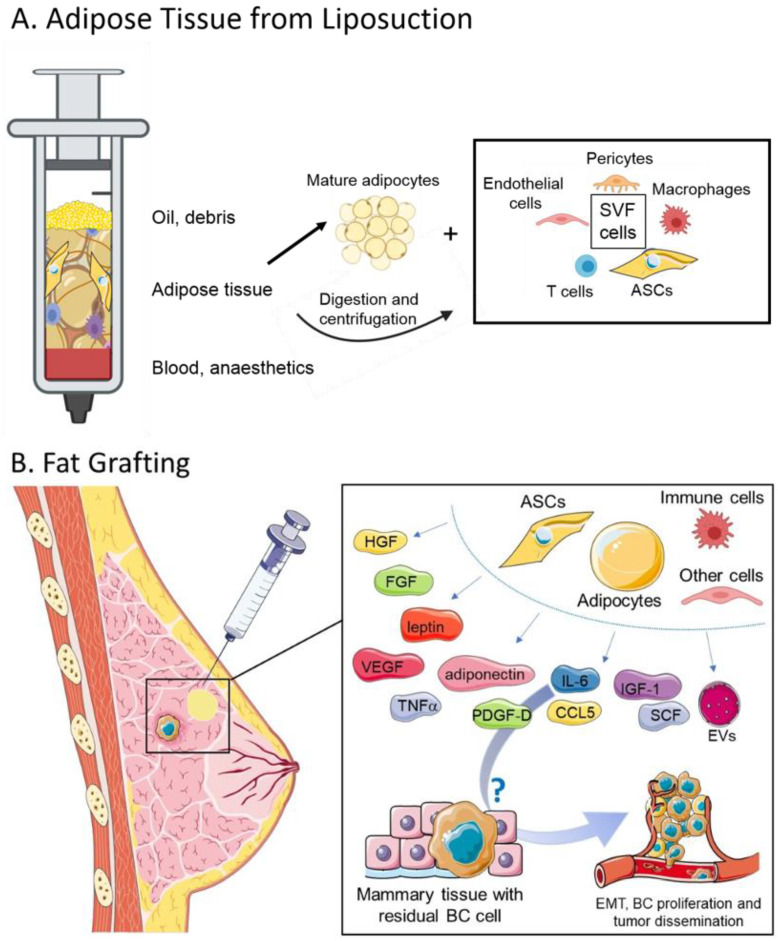
Lipofilling for breast reconstruction. (**A**) The liposuction syringe is centrifuged to separate purified fat from debris. Centrifuged lipoaspirate is separated into three phases: the oily fraction coming from disrupted adipocytes, watery fraction, which consists of blood, lidocaine, and saline injected before the liposuction, and the purified fat between the oily and watery fractions. Stromal vascular fraction (SVF) can be obtained from the whole adipose tissue by further digestion and centrifugation steps. (**B**) Harvested fat is injected into the mammary tissue. Several factors, such as growth factors, adipokines, and extracellular vesicles (EVs) are secreted by adipose-derived stromal cells (ASCs), adipocytes, and other cells of the adipose tissue, but if and how they could stimulate BC survival, progression, and metastasis is still debated.

Fat is usually processed by centrifugation, washing, or decantation. A widely used protocol to separate purified fat from debris is centrifugation. Once the fat has been collected, the fat syringes are centrifuged at 3000 rpm for 3 min. After centrifugation, the lipoaspirate is separated into three layers consisting of: the oily fraction coming from disrupted adipocytes; the watery fraction, which consists of blood, lidocaine, and saline injected before the liposuction; and the purified fat between the oily and watery fractions (Figure 1A) [20,30]. Once the purified fat is obtained, the breast skin is punctured under local anesthesia, by using an 18-gauge cannula that is also used to release dermato-fascial and scar tissue adhesions. The same cannula is then used to inject the fat graft in the subcutaneous and subglandular breast plane. The washing technique of fat processing is performed by washing with normal saline or 5% glucose solution [31] to remove blood, the oily fraction, and cellular debris from the aspirated fat [20]. A rare technique of fat processing is decantation, which uses gravity to separate the cellular component from the oily and watery one [20].

### 2.2. Cosmetic Success after Breast Surgery

LF leads to many benefits for its use in breast reconstruction, being able to correct volume defects with minimal scarring, improving esthetic results and leading to an increase in patient satisfaction rate [32]. Many clinical studies have been performed about LF as a first step in breast reconstruction for patients undergoing mastectomy and radiotherapy [33,34,35], while some of them were focused on patients receiving post-mastectomy radiotherapy only (PMRT) [36]. An analysis of 28 patients who underwent multiple LF sessions to prepare the thoracic region for breast implant reconstruction where FG was used as a surgical tool to improve the results of breast implant reconstruction after radiotherapy, in a case-control study, showed improved esthetic and functional outcomes, along with a reduced complication rate [33,37]. Two FG sessions during two-stage breast implant reconstruction ensured a lower rate of capsular contraction, although no statistical analysis was conducted [38]. The concept of LF has evolved as a primary tool in breast reconstruction. Deflation-LF sessions are conducted to prevent complications during reconstruction and reduce the frequency of postoperative complications. The positive effect of the deflation-LF procedure was demonstrated even with the removal of the expander at the end of the planned reconstruction, without any implant placement, in order to guarantee a totally autologous reconstruction [39,40,41]. A reconstructive system that includes prostheses and FG as integrated tools to improve the final reconstructive result with a volume consisting of fat and prosthesis in a variable percentage depending on the patient’s body profile and breast morphology did not show any complication rates higher than conventional two-stage reconstructions, achieving excellent esthetic results and high patient satisfaction levels [42]. Finally, in a case-control study on the long-term impact of LF in hybrid breast reconstruction, LF was significantly associated to reduction of capsular contracture rate, breast pain, and displacement/rotation of the implant and at long follow-up times, to lower the overall rates of revision surgery compared to standard expander-implant reconstruction. In the same study, patients treated with LF were significantly more satisfied with the appearance of their breast and showed an improved psychosocial, sexual, and physical well-being than patients exposed to prosthetic reconstruction alone [43]. It must be taken into account that data on esthetic outcomes investigating the efficacy and success of LF, measuring the gain in soft tissue thickness after surgery, mainly derive from case studies or retrospective studies. In fact, there are only a few conducted prospective studies which also involved small cohorts of patients. A substantial randomized controlled trial that compares a patients group receiving FG with a control group without any intervention is still missing.

Moreover, despite AFT showing a great variability in terms of surgical procedure and clinical outcomes, what all LF procedures have in common is the minimal complication at the donor site related to the liposuction step (such as hematoma formation or bruising). In contrast, a problem could be represented by the post-operative complications of the recipient site: in addition to pain, the injection of fat into poorly vascularized areas or the injection of large volume of fat into a single area may cause post-LF calcification, oil cyst or fat necrosis. Fat necrosis causes the formation of palpable masses which may require additional imaging and needle biopsy to complete post-operative monitoring [44].

### 2.3. Impact on Cancer Recurrence in BC Patients

Most of the published studies on LF are focused on technique, complications, fat graft survival, and cosmetic results, and only few of them deal with the risk of microcalcifications observed during the follow-up, affecting the radiological imaging interpretation [45,46,47]. More recent findings, on the contrary, suggest that LF is a procedure that does not affect radiological findings during follow-up in BC patients [48]. Moreover, it is well known that all kinds of breast surgery, including reduction, augmentation, and flap reconstruction, may induce fat necrosis, and therefore, calcifications [49,50,51]. In a prospective study about LF-induced appearance of normal and pathological imaging results, normal findings (“oil cysts”) were better identified using ultrasound, while liponecrosis, the most frequently observed pathological complication, was better detected by magnetic resonance imaging (MRI) compared to ultrasound and mammography [52].

Findings from experimental studies emphasized the role of adipocytes, the most abundant stromal cells, both in promotion and in protection against BC [53]. In the absence of translational research to demonstrate this clinical concern, the French Society of Plastic Surgery in 2007 addressed the question of cancer safety for the LF technique in BC patients, sending a recommendation to the French plastic surgeons to postpone LF in the breast with or without BC history unless it is performed under the prospective controlled protocol. Two years later, the ASPS Task Force for assessing the indications, the safety, and efficacy of AFT stated: “based on a limited number of studies with few cases, no interference with BC detection has been observed; however, more studies are needed” [45]. Despite the fact that post-lumpectomy and post-mastectomy are clearly included in the indications of FG, the Task Force did not discuss the issues of adipocytes-stroma interaction, and the risk of development of local recurrences.

In a retrospective study involving 321 consecutive patients operated for a primary breast LF, the technique appeared to be an overall safe procedure, even when patients undergoing quadrantectomy and mastectomy were analyzed separately or when the analysis was limited to invasive tumors. Just a higher risk of local events was observed when the analysis was limited to intraepithelial neoplasia in a small number of patients [54]. Clinical trial data collected in a large metanalysis suggest that these findings are limited to in vitro settings with no translation in vivo, for unclear reasons [55]. In 2407 patients, no evidence that LF significantly increases the risk of locoregional recurrence was found when used as part of a breast reconstruction procedure. A Matched Controlled Study on patients undergoing segmental or total mastectomy for BC or BC risk reduction or benign disease as well, followed by breast reconstruction involving LF, reported that no increase in rates of locoregional recurrence, systemic recurrence, or second BC were seen, supporting the oncologic safety of LF in breast reconstruction. Only in patient under hormonal therapy the risk of locoregional recurrence of BC was slightly higher, even if the recurrence rates were low, in people who received LF compared with patients who did not receive LF. However, this observation did not support anyway the contraindication of LF as a reconstructive option for BC patients receiving hormonal therapy [56]. LF showed to be a safe procedure even in a small subset of high-risk population, such as BRCA mutation carriers, that have aggressive disease with a higher risk to develop second tumor. However, the number of involved patients and the follow-up length do not allow to draw final conclusions [57].

## 3. Adipose Tissue: Composition and Functions

### 3.1. Composition of the Adipose Tissue

Adipose tissue (AT) is a multicellular and non-homogenous endocrine organ composed of adipocytes, preadipocytes, fibroblast, immune cells, endothelial cells, and adipose stem/stromal cells, each having an undeniable role in human health and disease. Of note, adipokines are peptides secreted by all AT resident cells including endothelial and immune cells [58,59]. As evidenced by the most comprehensive study of AT composition to date, the heterogeneity of fat tissue is even more complex than previously considered and included unexpected cell populations such as smooth muscle cells, chondrocytes, and osteoblasts [60]. Moreover, the significant differences in cell populations observed between AT depots, gender, body mass index (BMI), as well as across stages of type-2 diabetes indicated that the dynamic heterogeneity of AT might play the leading role in health and disease process [60]. The adipose organ is composed of numerous discrete anatomical depots. Most of human body fat is located just under the skin, mainly in the abdominal, subscapular, gluteal, and femoral areas, and thus, it is referred to as subcutaneous adipose tissues (SAT). Intra-abdominal fat surrounding digestive organs is called visceral adipose tissue (VAT) [61]. Importantly, although VAT makes up only a small proportion of body fat, it is a key player in a variety of health problems, such as hepatic insulin resistance and type 2 diabetes. Apparently, because VAT is hormonally active tissue and drains its secretes into the portal vein which supplies blood flow to the liver, VAT accumulated in obesity is particularly hazardous for the liver. Contrary to VAT, SAT, due to its reduced lipolytic rate and strong avidity for free fatty acids (FFAs), regulates the central adiposity, as removal of thigh fat by liposuction was followed by AT re-accumulation and redistribution preferentially in the abdominal space and protects liver from excessive FFAs deposition characteristic for metabolic syndrome [62,63,64]. Women, when compared to men, have higher percent of body fat; however, the different pattern of its distribution seen in a woman’s body (in subcutaneous gluteal-femoral fat) is associated with lower metabolic risk [65], although women with so-called upper body obesity (evidenced by a waist-to-hip ratio >0.85) suffer from the same metabolic complications as men [66]. Interestingly, the differences in AT depots go beyond straightforward division SAT vs. VAT, as it has been recently demonstrated that abdominal SAT is characterized by smaller adipocytes and a peculiar pattern of gene expression compared to femoral AT in overweight/obese women [67]. Paired comparison of the proteome of human abdominal and femoral SAT derived from metabolically compromised individuals following an overnight fast, indicated significant differences in the expression of 22 proteins mostly related to cellular structure, including the extracellular matrix (ECM). More detailed examination indicated ~25% higher expression of periostin (POSTN) in femoral AT when compared to abdominal AT [68]. Periostin is a secreted protein, important in tissue development and regeneration, including wound healing [69]; however, through binding to integrins, periostin supports adhesion and migration of epithelial cells playing a role in cancer stem cell (CSC) maintenance and metastasis [70,71].

### 3.2. ASCs and the Regenerative Property of Fat Grafting

SAT represents an abundant and accessible source of multipotent stem cells responsible for the regenerative potential of FG used not only to correct soft-tissue defects after breast reconstruction but including different clinical applications [44,72]. Human AT was described as source of adult mesenchymal stem cells (MSCs) for the first time by Zuk et al. in 2001 [73]. Adipose-derived stromal cells (ASCs) is the official term established by the International Fat Applied Technology Society (IFATS) describing non homogenous multipotent population of any plastic-adherent, stable-doubling cells derived from lipoaspirate [74]. ASCs are localized in the stromal vascular fraction (SVF), which constitutes a heterogeneous population of cells including fibroblasts, hematopoietic-lineage cells, endothelial cells, pericytes, and preadipocytes (Figure 1A). SVF has regenerative properties due to paracrine effects: secretion of growth factors, chemokines, cytokines, and promotion of angiogenesis. It also has immunomodulatory properties due to the production of mediators of inflammation and cell-adhesion molecules. Stromal and vascular cells of the SVF, including ASCs, possess a developmental in vitro plasticity (expansion by adherent culturing), which can be obtained by different standard protocols (enzymatic or mechanical isolation methods), and are characterized by the expression of specific cell surface markers. ASCs can be isolated from other SVF cells, such as blood and immune cells, using multicolor flow cytometry [75]. In 1966, Rodbell et al. proposed an enzymatic method to isolate cell from rat AT, obtaining floating mature adipocytes on the top separated from pelleted SVF [76]. Established by International Society for Cellular Therapy (ISCT) in 2006 hallmark ASCs antigens included: CD73, CD90, and CD105 with concomitant lack of expression of CD45, CD34, CD14 or CD11b, CD79a or CD19, and HLA-DR surface markers [77]. Of note, freshly isolated ASCs express CD34 but after 8–12 passages in vitro, they do not express CD34 anymore [78]. In 2013, the IFATS defined ASCs as CD44-, CD73-, CD90-, and CD105-positive, while CD31- and CD45-negative [74]. Additionally, the expression of CD36 and lack of CD106 distinguish ASCs from bone marrow MSCs [74]. However, there is still a lack of consensus in definitive markers that discriminate ASCs [79], because surface biomarkers differ depending on donor sites and culture procedure [80].

ASCs show the same multi-lineage differentiation potential (osteogenic, adipogenic, myogenic, and chondrogenic) and the capability to self-renew of other MSCs [5,6]. Differentiation and self-renewal capacities of ASCs, and thereby, their usefulness in breast reconstruction highly depends on donor age and AT depots. ASCs derived from breast fat and SAT are similar in cell phenotype and genetic characteristics [81]. Thus, referring to breast reconstruction, abdominal SAT taken from young donors is the most relevant source of somatic stem cells [80]. Compared to MSCs from umbilical cord and bone marrow, ASCs have similar morphology and immunophenotypes but higher adipogenic capacity. This different adipogenic potential was significantly correlated by Liu et al. to the expression pattern of the axis miR-301 b ~ miR-130 b—PPARγ [82]. Adipogenesis can be induced in vitro using standard supplements such as IBMX (isobutyl methylxanthine), indomethacin, insulin and dexamethasone in different concentrations obtaining the differentiation of ASCs after almost 14 days [82,83,84]. The ability of ASCs to undergo differentiation into multiple cell lineages, together with their capacity to reduce apoptosis, modulate the immune response, and promote angiogenesis by secreting growth factors and cytokines following various stimuli (especially hypoxia), means that they can be used for different clinical purposes [85,86,87,88]. ASCs also show a protective role in patients undergoing allogenic stem cell transplantation preventing GVHD (Graft Versus Host Disease) [89]. In light of this, for their plasticity and differentiation potential, the easily accessible ASCs seem to be particularly promising in tissue engineering and regenerative medicine. Despite these successes, a major challenge still persists: even though FG has demonstrated to be extremely useful for the treatment of soft tissue defects and different procedures have been developed to improve the survival of transplanted fat graft, inconsistent and unsatisfactory outcomes often occur observing an average reduction in breast volume over time from 20% to 90% and requesting additional sessions [90]. The cause might be the fragility of mature adipocytes and the fact that they are little resistant to the insults connected to fat harvesting, processing, and injection. In contrast to adipocytes, preadipocytes are able to better survive in hypoxia and ischemic conditions [91]. In this regard, adipose-derived regenerative cell (ADRC)-enriched FG has been shown to increase fat graft retention in animal models [92]; additionally, in clinical trials its efficacy and safety were demonstrated, thus suggesting it as a reconstructive option for women with post-BCS defects [93]. ASCs isolated from lipoaspirate are purified, concentrated, or expanded ex vivo, and then added to the fat graft tissue before injection. Pre-enriched fat graft with ex vivo expanded autologous ASCs has been demonstrated to reduce fat reabsorption in patients with contour deformities [94] and to improve breast volume in retention in patients undergoing surgery for breast augmentation [95]. Despite further additional studies and clinical trials being needed, available data seem to demonstrate the efficacy and safety of ASCs supplementation in order to obtain better results and improvements in FG.

## 4. Adipose Tissue and BC Crosstalk

The epidemiological and etiological evidence showing that obesity is a significant risk and negative prognosis factor for BC [96] has oriented the interest of researchers to better understanding of the AT potency to enhance tumorigenic growth activity of cancer cells. A special role in cancer progression has been attributed to mature adipocytes, key components in the stroma of BC. Breast AT is necessary for correct morphogenesis of mammary glands. Leptin and adiponectin signaling pathways have a very special role in this process [97]. In the past, adipocytes were considered as terminally differentiated cells [98], but now we know that due to physiological interaction with mammary alveolar cells, they undergo repeated cycles of de-differentiation and re-differentiation [99]. Adipocytes are fuel reservoir and may serve fatty acids (FAs) and glycerol due to basal and stimulated lipolysis [100]. However, lipid trafficking between adipocytes and epithelial cells has also been implicated in BC metastasis [101]. In fact, beyond FAs, AT releases: (i) adipokines such as leptin, adiponectin, and resistin, (ii) growth factors (IGF1, insulin-like growth factor 1; VEGF, vascular endothelial growth factor; EGF, epidermal growth factor; FGF, fibroblast growth factor; HGF, hepatocyte growth factor; NGF, nerve growth factor; TGFβ, transforming growth factor), (iii) enzymes (autotaxin), (iv) cytokines (interleukin [IL]-1, IL-6, IL-8, C-C motif ligand 5-CCL5, tumor necrosis factor-TNF-α), (v) ECM proteins (collagen VI and its cleavage product endotrophin), and (vi) exosomes [102] within local and entire organism range. To date, some of these active molecules, besides contributing to physiological cell–cell communication, have been reported to promote BCs’ broadly understood aggressive phenotype i.e., cell proliferation, motility, invasiveness, epithelial to mesenchymal transition (EMT), and stemness, but also tumor angiogenesis [97]. Therefore, it becomes obvious that some physiological characteristics of AT and mechanisms governing such a particular dialog between adipocytes and epithelial may be employed by BC cells to satisfy their stimulatory, supportive, and nutritive needs. The hallmarks of healthy AT are as follows: (i) a high degree of vascular density, (ii) minimal level of hypoxia and fibrosis, and (iii) a low level of ‘M1′ macrophage infiltration and low level of inflammation [103].

Adipocytes involved in different pathologies such as obesity and BC share common dysfunctional phenotype, distinct from described above “healthy phenotype” and have detrimental effects when it comes to tumor progression and patient prognosis [104]. Obesity, defined as an excessive body fat, has been associated both with a higher risk of developing BC, particularly in postmenopausal women, and with worse disease outcome for women of all ages [105], and elevated waist-to-hip ratio (WHR) was as a predictor of BC mortality in estrogen receptor (ER)-positive postmenopausal women [106]. Excessive AT expansion causes hypoxia, escalating mechanical stress and ECM deposition, and infiltration of immune cells (mainly proinflammatory macrophages), followed by chronic low-grade inflammatory condition that strongly impacts BC [107,108]. The enlarged adipocytes release more of FFAs and together with infiltrating immune cells are the primary sources of many inflammatory proteins, including CCL2, TNF-α, IL-6, IL-18, leptin, resistin, plasminogen activator inhibitor (PAI)-1, visfatin, retinol binding protein 4 (RBP4), and angiopoietin-like protein 2 (ANGPTL2), contributing to tumor progression. In contrary, the expression levels of anti-inflammatory and insulin-sensitizing molecules such as IL-10, adiponectin and secreted frizzled-related protein 5 (SFRP5) are substantially reduced in AT in obesity [109]. Moreover, obese AT releases high amounts of extracellular vesicles (EVs), which target BC cells and promote malignancy [108,110].

BC-neighbored aberrant adipocytes are named cancer-associated adipocytes (CAAs) and they are found in the tumor invasive front [111]. When compared to mature “naïve” adipocytes, CAAs are smaller in size and contain less lipids in dispersed lipid droplets. Furthermore, CAAs are characterized by decreased expression of adipogenesis markers (dedifferentiated phenotype) and overexpression of inflammatory cytokines [111]. Although in vitro models (cancer cells/adipocytes) confirmed that phenotype of CAAs may be triggered directly by cancer cells [112], and some candidate molecules released by tumor cells such as TNF-α, Wnt3a, Wnt5a, and MMP11 have been proposed, the exact mechanism remains elusive [104]. In addition to paracrine factors released by cancer cells, physical changes (increased compression) experienced by the tumor stroma during rapid expansion of solid tumors can induce adipocyte de-differentiation via mechanically activating Wnt/β-catenin signaling, in turn contributing to the enhanced tumor growth [113]. Like adipocytes in obesity, CAAs secrete significantly higher levels of motility factors such as CCL2, CCL5, autotoxin (ATX), as well as proinflammatory cytokines such as IL-1β, IL-6, TNF-α, VEGF, insulin-like growth factor binding protein-2 (IGFBP-2), and leptin but much less of adiponectin [104]. All these features have changed the perspective and CAAs are no longer considered only as the passive companion, but rather as the lead character promoting the invasion and metastasis of BC [102,114].

Taking for example adiponectin and leptin clearly reveals the ambiguous effect of AT secretes on tumor progression. Adiponectin is mainly secreted by AT and exerts different biological functions globally improving glucose and lipid metabolism [115,116]. Physiologically, adiponectin in one of the most abundant proteins present in the systemic circulation, contributing to 0.05% of the total proteins [117,118]. Low adiponectin levels have also been linked to an increased risk of different malignancies including BC [119,120]. Furthermore, recent study proved that adiponectin levels may be useful to predict survival rates in BC or may be used as a marker/predictor for defining patients who require more aggressive treatment [121]. Adiponectin binds to adiponectin receptors AdipoR1 and AdipoR2, each expressed in histologically normal and malignant breast tissues [122,123]. In BC adiponectin exerts its function through activation of numerous signaling pathways, e.g., (i) decreases the phosphorylation of PI3K and Akt, (ii) induces AMPK activation suppressing mTOR pathway, (iii) induces cell cycle arrest through the downregulation of cyclin D, thus preventing breast tumor growth [124], and (iv) negatively impact lamellipodia formation, thus preventing cell migration and invasion [118]. Importantly, the mentioned anti-proliferative and pro-apoptotic effects of adiponectin were seen in mostly ERα-negative BC, while divergent effects of adiponectin were reported in ERα-positive BC such as: (i) activation of both MAPK and Akt stimulating mTOR/p70S6K cascade, (ii) activation of ERα followed by recruitment of LKB1 as a receptor coactivator overall compromising AMPK and in consequence switching cancer cell energy balance vs. lipogenic aggressive phenotype, and (iii) upregulation of cyclin D1 promoting tumor growth [125]. Leptin is a hormone discovered almost 30 years ago [126]. Today, we know that beyond central role in maintaining energy homeostasis, leptin participates in the regulation of pathological processes such as cancer, by having mitogenic, antiapoptotic, and proinflammatory activity. Prevalently, leptin exerts its biological function through binding to its receptor (ObR), which activates multiple downstream signaling pathways, such as JAK2-STAT3, MAPK, and PI3K/AKT, involved in tumorigenesis from initiation, growth to metastatic progression, and chemoresistance [127,128]. Additionally, in triple negative BC (TNBC), leptin receptor plays the key role in CSC self-renewal and tumorigenicity, as no tumors were formed in mice transplanted with leptin receptor-silenced cells [129,130].

Similarly to adipocytes, ASCs can also be “educated” by cancer cells, and consequently, secrete factors implicated in BC survival, progression, metastasis, and chemo-resistance, such as CXCL1, CXCL2, CCL5, SDF1, IL-6, IL-8, TGFβ, and miRNA containing macrovesicles (miRNA-21 and miRNA-34a) [131,132,133]. Very recent study evidenced that ASCs can fuse with BC cells spontaneously forming hybrid cell with higher than parent cell tumorigenic potential [102,134]. Taken together, these data indicate that ASCs might be largely modified by cancer cells and, once educated, may become danger partners in tumor progression.

## 5. Oncologic Safety of Breast Lipofilling: A Still Debated Issue

Considering that AT is a rich source of growth factors and that an increasing body of evidence shows an intense crosstalk between ASCs and cancer cells, the oncologic safety of LF procedure has been questioned. Yet, it remains unclear whether the AT derived from lipoaspirate really have the ability to stimulate BC growth and progression (Figure 1B). The most recent pre-clinical studies investigating the role of human lipoaspirate on BC are described below and summarized in Table 1.

### 5.1. Effect of Lipoaspirate on BC Cells In Vitro

In 2017, Almarzouqi et al. analyzed the effects of lipoaspirate on BC cells, showing a significant increase in the proliferation rate of MCF-7 cells co-cultured for 5 days with lipoaspirate and compared to the controls lacking AT [135]. Co-culture with AT also triggered increased expression of matrix metalloproteinase (MMP)-1 and integrin α2, indicating increased capability of the tumor cells to degrade the ECM, suggesting a higher tendency to form metastasis upon interaction with AT. A previous study from Massa et al. also reported co-culture experiments using BC cell lines (MDA-MB-231, MCF-7, ZR-75) and human tissue from lipoaspirate [136]. Obtained results showed that all cancer cell lines displayed a significant increase in the proliferation rate when co-cultured in the presence of either whole AT or in vitro-differentiated adipocytes, thus suggesting a significant pro-tumorigenic effect related to the presence of AT.

Differently, Rowan et al. used isolated human ASCs from abdominal lipoaspirate from healthy donors and examined their pro-tumorigenic potential on MDA-MB-231 model of TNBC [137]. ASCs, either co-cultured with MDA-MB-231 or used to generate a conditioned medium (CM) for tumor cell stimulation, did not enhance cancer cells growth in vitro. However, indirect co-culture with ASCs as well as ASCs-CM stimulated migration of MDA-MB-231 cells, thus suggesting that paracrine factors released by ASCs trigger an effect on cancer cells. Similar results were obtained by Kucerova et al., who investigated the effect of ASCs from lipoaspirate on the HER2-overexpressing, estrogen/progesterone receptor-negative SKBR3 cells [138]. They observed that ASCs and ASCs-CM did not increase BC cells proliferation. However, they significantly increased tumor cell migration, mammosphere formation, and EMT, as demonstrated by the upregulation of Nanog, Oct, Twist, Snail1, Snail2, αSMA, and FAP in SKBR3 cells exposed to ASCs-CM. At the same time, ASCs and secreted soluble factors increased the chemosensitivity of SKBR3 cells to doxorubicin and 5-fluorouracil, further showing that tumor and adipose cells interact in a complex fashion and are able to alter tumor cells properties and drug responsiveness. In another study, ASCs were reported to induce a mesenchymal switch in BC cells through a paracrine manner. T47D, MCF-7, and BT-474 BC cells incubated with ASCs-CM showed reduced expression of E-cadherin and upregulation of EMT markers such as Slung, Snail, α-SMA, fibronectin, and vimentin [139]. In the same study, ASCs-CM also promoted cancer cell colony formation. This effect was inhibited in presence of platelet-derived growth factor (PDGF)-D neutralizing antibodies, suggesting a possible mechanism for ASCs-dependent pro-tumorigenic effect where PDGF-D pathway is critical. Another study reported that EVs secreted by human ASCs promoted migration of MCF-7 cells through activation of the Wnt/β-catenin signaling pathway [140]. To further explore the molecular mechanisms underlying the effect of ASCs on BC cells migration, Xu et al. analyzed the levels of several proteins and soluble factors in direct co-culture between murine 4T1 BC cells and ASCs from inguinal fat [148]. They found that ASCs-released stem cell factor (SCF) plays a crucial role in ASCs-induced BC migration and invasion, through the downregulation of miR-20b and the subsequent SCF-dependent induction of the MAPK-p38/E2F1 cascade in BC cells. Moreover, they showed a positive correlation between ASCs-released SCF and HIF-1α/VEGFA, two targets of miR20b that may drive BC cell migration and invasion upon stimulation with ASCs. Certainly, human tissue-derived cells are needed to prove the relevance of such a mechanism in real patients. Moreover, the huge heterogeneity of BC raises a concern on whether a common mechanism shared by all BC cells may be identified.

Interestingly, Eterno et al. pointed out that not all BC cells are similarly susceptible to the presence of ASCs. They combined autologous ASCs from lipoaspirate and BC cells isolated from the same human donors in order to evaluate their potential interactions [141]. When co-cultured in a transwell system, two out of four samples showed increased cancer cell migratory capability in presence of ASCs-CM. By contrast, the other two samples did not show enhanced migration upon ASCs co-culture. ASCs could not chemoattract normal mammary epithelial cells, which did not show any migratory potential upon co-culture. In the same study, ASCs derived from all four donors were proved to increase aggressiveness of MDA-MB-231 cells (as shown by enhanced migration and upregulated expression of twist1, snail1, and vimentin), while they did not affect the behavior of MCF-7 cells. These observations suggest that ASCs may not be able to influence the tumorigenic potential of all BC cells. The authors found that BC cells susceptible to ASCs expressed higher levels of the proto-oncogene c-Met as compared to those cells insensitive to ASCs. Therefore, susceptibility to ASCs may be more associated to specific BC features than to intrinsic pro-tumorigenic properties of ASCs. In turn, when co-cultured with c-Met expressing cells, ASCs expressed higher levels of the mitogen HGF, thus suggesting a double ASCs-cancer cell crosstalk mediated through HGF/c-Met signaling.

In 2019, Wu et al. isolated human ASCs from SAT of patients undergoing liposuction and demonstrated that ASCs-CM significantly reduced viability of MCF-7, MDA-MB-231, and MDA-MB-468 BC cells in a dose-dependent manner [142]. In particular, treatment of BC cells with ASCs-CM rapidly induced ATM/Chk2 dependent DNA damage response, cleavage of caspase 3 and apoptosis in BC cells but not in non-cancerous mammary cell line. The most potent effect was observed in MCF-7 cells.

It has to be noted that all the above-mentioned studies have some limitations. Firstly, the in vitro nature of the used system, which does not completely reflect the breast microenvironment. Secondly, a limited set of samples used, meaning that the AT from few patients was tested for analyses, thus raising some concerns about the robustness and universal applicability of the results.

### 5.2. In Vivo Studies on Animal Models of BC

To gain more translational insights into the potential risks of LF in BC, some pre-clinical studies have explored the effects of FG in animal models, but they have also obtained mixed conclusions (Table 1).

In 2013, Orecchioni et al. isolated human white AT from patients undergoing breast reconstruction and either used it unfractionated or sorted to separate endothelial progenitors (CD34+ CD31+) and ASCs (CD34+ CD13+) from mature pericytes/fibroblasts (CD34-) [143]. In this study, AT as a whole or as sorted cellular components was co-injected with MDA-MB-436 or HCC1937 BC cells in the mammary fat pad of immunodeficient mice. Co-administration of AT, endothelial component, or ASCs significantly increased tumor volume, while CD34- population did not show any effect over control mice. The presence of CD34+ white AT also increased the number of lung metastases in mice undergoing mastectomy. Accordingly, in vitro investigations revealed that endothelial cells and ASCs from human AT were the main responsible for the overexpression of genes involved in EMT in BC cells. In particular, the induction of EMT was more relevant in luminal cells as compared with TNBC line.

Other studies focused on the role of ASCs isolated from human fat and co-implanted with tumor cells into the mammary fat pad of mice. In a first paper, the authors showed that ASCs isolated from a patient with BMI 18.3 were able to stimulate the growth of MDA-MB-231 xenografts [137]. Such tumors displayed elevated vimentin, MMP-9, IL-8, VEGF, and CD31, pointing out the potential of co-injected ASCs to promote EMT and angiogenesis. On the contrary, when ASCs derived from a patient with BMI 25.0 were co-injected with tumor cells, no difference in tumor volume was observed over 40 days of growth, suggesting that the pro-tumorigenic role of ASCs may be donor dependent. Both co-injection groups exhibited metastases to the lung and liver not evident in mice injected with MDA-MB-231 alone. A second study also reported a sample-dependent tumorigenic effect of ASCs. In this case, lipoaspirate-derived ASCs and BC cells were isolated from four human donors and co-injected subcutaneously in immune-compromised mice. Only half of the mice developed a tumor, while in the others, ASCs did not promote tumorigenesis of combined cancer cells [141]. Moreover, human ASCs from all four different donors were tested in combination with two different BC cell lines. It was found that ASCs enhanced the tumorigenic potential of MDA-MB-231 in vivo, inducing formation of bigger and highly proliferating tumors as compared to mice injected with tumor cells alone. By contrast, the same four samples of ASCs failed to enhance tumorigenicity of MCF-7 cells, whose behavior was not altered by the presence of ASCs. Therefore, it seems that ASCs are not tumorigenic per se, but rather, they could favor tumor growth and aggressiveness in specific BC subtypes.

Interestingly, in Tsuji et al., two approaches were investigated to either study the case of concomitant engraftment of BC cells and AT or model breast reconstruction in case of residual BC cells [144]. In the first approach, increasing doses of MDA-MB-231 and BT-474 were seeded into human fat graft derived from patients undergoing elective surgery, and mixed before subcutaneous implantation in mice. At six weeks after injection, histological assessment revealed that BT-474 seeded into lipograft did not engraft at any dose, while they did in the Matrigel control. MDA-MB-231 mixed within lipograft were able to engraft at a cell dose one order of magnitude higher than in Matrigel, thus suggesting that AT is a less favorable environment for tumor cells engraftment as compared to Matrigel. In the second approach, cancer cells were engrafted and human fat was injected after two weeks at the same site of tumor cells. At the time of euthanasia, pan-cytokeratin, and Ki67 staining showed decreased tumor cell proliferation index in lipoaspirate groups associated with both MDA-MB-231 and BT-474 cell lines. Similarly to this second approach, Silva et al. examined the impact of human-derived lipoaspirate grafted in a humanized mouse model of MCF-7-induced BC [145]. In this study, AT was obtained from a 57-year-old woman undergoing elective abdominoplasty and FG was performed in mice two weeks after MCF-7 xenografting. Results showed that the tumor volume was reduced in the group receiving FG as compared to saline control group. Accordingly, Ki67 expression performed on tumor sections ex vivo was lower upon fat injection. Hypothesis for such observations may be found in the fact that AT may cause mechanical compression in the tumor mass, thus reducing cancer cell proliferation but also inducing tumor hypoxia [149]. It is also possible that some components of the transferred AT may have antitumorigenic effects, but this is still to be investigated and proved [150].

## 6. Open Questions

LF methods are clinically favorable in terms of long-term stability of the results and have become frequently used by plastic surgeons dealing with breast reconstruction after mastectomy or BCS [151]. However, patient safety must have priority when programming such an intervention for oncological patients. Results obtained in pre-clinical studies so far showed contradictory and non-homogeneous results, in part suggesting a pro-tumorigenic role of LF, in part indicating that it is associated with the downregulation of proliferation in local tumor cells.

Facing such a complicated scenario, an aspect to be considered is the heterogeneity of cell populations inside the AT. Mature adipocytes, ASCs, and endothelial and fibroblast cells are key components of lipoaspirate; different materials have been used in different studies and they may have had an impact on observed results. Quite recently, it has been reported that the ASC.B6 cell line upregulates IGF1 and downregulates the IGF1 regulator IGFBP2, thus generating a strong pro-mitogenic signal resulting in enhanced tumor growth. Differently, freshly isolated ASCs express and secrete huge amount of IGFBP2, which inhibit Akt phosphorylation and proliferation of cancer cells [152]. Accordingly, ASC.B6 cells were shown to promote BC cell proliferation better than normal fat-derived ASCs, suggesting that they gained a stronger tumor-promoting function. Therefore, the results observed in some studies with immortalized cell lines or ASCs grown in culture for several passages may have suffered from a bias and may not represent the real state of ASCs, whose gene expression changes with the time in culture [153].

Moreover, biological material from different patients may be different. Pre-clinical studies performed so far have denoted a different behavior when using different donors of AT as well as different types of BC cells. This aspect deserves major attention, as it strongly suggests that the risks and safety of LF may not be universally stated. For example, it is known that obesity impacts not only mature adipocytes but also ASCs. Recent studies have evidenced that high levels of leptin produced by obesity-altered ASCs derived from human lipoaspirate of SAT isolated from obese women promote TNBC cell migration and metastasis as well through induction of EMT process [146] as well as growth and metastasis in ERα-positive BC [147]. Therefore, the utility and most of all safety of lipoaspirates derived from donors with obesity in LF procedure should be carefully examined. Additionally, the state of BC and its biological/molecular/genetic portrait seem to play a role in the susceptibility to AFT. The inclusion of different BC types in vitro and in vivo could yield the contradictory and not homogeneous obtained results. The classification by immunohistochemistry or gene expression profiling in different molecular subtypes of BC (Luminal A, Luminal B, TNBC, HER2-enriched) represents a critical component in the disease characterization, providing information about prognosis and influencing treatment planning. The different molecular subtypes may play a role in disease recurrence, treatment response and outcomes. For example, ER signaling pathways fosterERα-positive BC tumor progression [154] and the AT paracrine effect could amplify this pro-tumorigenic effect suggesting a different crosstalk in ERα-positive BC upon AFT. However, no clinical study has significantly demonstrated the impact of AFT on locoregional recurrence or distant metastasis in relation to BC molecular subtypes probably because of unbalanced stratifications in different subtypes or no sufficiently long follow-up times. In 2019, Sorrentino et al. reported a possible increase in late locoregional recurrence for Luminal A patients treated by AFT [155]. Despite this intriguing finding, it should be noted that only less than one-third of the initial Luminal A population was still on follow-up after 80 months, being aware of the correlation of Luminal cancers to late recurrences. To date, clinical studies that stratify BC into their molecular subtypes with adequate powering are missing but required to make conclusions about the potential impact of LF after BCS on cancer recurrence. Additionally, further studies with longer post-operative follow-up and larger patient and matched control groups are required to address the safety of LF and prospective studies are needed.

In context of LF, the interaction of BC cells with AT resident MSCs should be taken into account for its potential role in BC progression. Especially after BCS, the remaining mammary tissue may be altered by the previous presence of BC, being more prone to pro-tumorigenic behaviors [156]. Preclinical studies tracking the evolution of ACSs injected in a breast where a tumor was previously growing will deliver useful information in the field.

Finally, the recent data demonstrated that enrichment with ex vivo-expanded ASCs-graft might improve conventional LF procedures, mainly due to reduced resorption rate, obtained presumably because ASCs are more resistant the hypoxia than mature adipocytes, and thus, survive better in grafted tissue prior to new vessels formation [95]. However, ASC-enriched fat grafting requires a liposuction procedure prior to the primary operation and ex vivo cell expansion requires suitable and costly facilities as well as expertise. In this regard, application of this method in clinical practice needs to be justified by superior results/maximum patient safety. The extensive studies are needed to understand the proangiogenic, anti-apoptotic, and immunomodulatory properties of ASCs expanded in vitro and whether they may act as tumor promoters. Here, an additional question opening new scientific paths arises, namely, whether the use of allogenic ASCs as off-the-shelf product will be possible in clinical practice?

## 7. Conclusions

Several studies have attempted to address the oncological safety of LF. Most clinical evidence has reported that LF does not induce increased risk of BC recurrence; however, all of them concluded with a recommendation for future studies to further investigate safety. Pre-clinical in vitro and in vivo research deserves attention in the field, as it revealed potentially alarming effects of AT-BC crosstalk. The clinical significance of this research is still unclear and prompts scientists to develop relevant models more accurately representing the real situation of LF after breast oncological surgery. Further studies should be done in order to better characterize the tumorigenic activity of lipoaspirate components and to define predictors of cancer recurrence after LF in post-surgery BC patients.

## Figures and Tables

**Table 1:** Most recent pre-clinical studies (last 10 years) investigating the role of human lipoaspirate on BC.

Reference	Study Setting	AT Component	BC Model	Results
Almarzouqi et al. [135]	In vitro	Whole AT	MCF-7 cells	Increased BC proliferation rate; triggered expression of MMP1, integrin α2
Massa et al. [136]	In vitro	Whole AT; in vitro differentiated adipocytes	MDA-MB-231, MCF-7, ZR-75 cells	Increased BC proliferation
Rowan et al. [137]	In vitro, in vivo	ASCs	MDA-MB-231 cells	Stimulated BC migration; stimulated tumor growth by ASCs from BMI 18.3 patient but not BMI 25.0 patient
Kucerova et al. [138]	In vitro	ASCs	SKBR3 cells	Increased BC migration, mammosphere formation, EMT, chemosensitivity
Devarajan et al. [139]	In vitro	ASCs	T47D, MCF-7, BT-474 cells	Induced EMT; promoted PDGF-D mediated colony formation
Lin et al. [140]	In vitro	ASCs	MCF7 cells	Promoted BC migration through ASCs-EVs
Eterno et al. [141]	In vitro, in vivo	Autologous ASCs	BC cells from human donors; MDA-MB-231, MCF-7 cells	Enhanced aggressiveness in MDA-MB-231 and in 50% of patient-derived BC
Wu et al. [142]	In vitro	ASCs	MDA-MB-231, MDA-MB-468, MCF-7 cells	Reduced BC viability
Orecchioni et al. [143]	In vitro, in vivo	Whole AT, ASCs, endothelial progenitors	MDA-MB-436, HCC1937 cells	Induced EMT; increased tumor volume and lung metastases
Tsuji et al. [144]	In vivo	lipograft	MDA-MB-231, BT-474 xenografts	Reduced engraftment; decreased BC proliferation
Silva et al. [145]	In vivo	Whole AT	MCF-7 xenograft	Reduced tumor volume and Ki67
Sabol et al. [146]	In vitro, in vivo	obASCs	TNBC cell lines; TNBC patient-derived xenograft	Promoted metastasis through leptin signaling
Sabol et al. [147]	In vitro, in vivo	obASCs	MCF7-Y537S cells; PDX models WHIM20 (Y537S mutation) and WHIM43 (D538G mutation)	Promoted metastasis in BC with mutant ERα; in vitro obASCs promoted proliferation and migration of ER WT and ER MUT cells

AT, adipose tissue; ASCs, adipose-derived stromal cells; BC, breast cancer, EMT, epithelial–mesenchymal transition; EVs, extracellular vesicles; obASCs, obesity-altered adipose stem cells; WAT, white adipose tissue.

## Data Availability

No new data were created or analyzed in this study. Data sharing is not applicable to this article.
